# Mesoscopic Simulation of the Two-Component System of Coupled Sine-Gordon Equations with Lattice Boltzmann Method

**DOI:** 10.3390/e21060542

**Published:** 2019-05-28

**Authors:** Demei Li, Huilin Lai, Chuandong Lin

**Affiliations:** 1 College of Mathematics and Informatics, FJKLMAA, Fujian Normal University, Fuzhou 350007, China; 2 Sino-French Institute of Nuclear Engineering and Technology, Sun Yat-Sen University, Zhuhai 519082, China

**Keywords:** lattice Boltzmann method, coupled sine-Gordon equations, Chapman-Enskog expansion, nonlinear partial differential equations

## Abstract

In this paper, a new lattice Boltzmann model for the two-component system of coupled sine-Gordon equations is presented by using the coupled mesoscopic Boltzmann equations. Via the Chapman-Enskog multiscale expansion, the macroscopical governing evolution system can be recovered correctly by selecting suitable discrete equilibrium distribution functions and the amending functions. The mesoscopic model has been validated by several related issues where analytic solutions are available. The experimental results show that the numerical results are consistent with the analytic solutions. From the mesoscopic point of view, the present approach provides a new way for studying the complex nonlinear partial differential equations arising in natural nonlinear phenomena of engineering and science.

## 1. Introduction

It is well known that most of the nonlinear phenomena that arise in engineering fields and mathematical physics, including plasma physics, fluid dynamics and nonlinear fiber optics, can be described by nonlinear partial differential equations (NPDEs). NPDEs have become an available tool for describing these natural nonlinear phenomena of engineering and science models. Hence, it becomes more and more important to be acquainted with all traditional and recently developed methods for NPDEs, and implementation of these methods [1,2]. Some of the most interesting features or physical rules are concealed in their nonlinear characteristics and can only be researched with an approximate method that is designed for inherent nonlinearity issues. As a result of the complexity and nonlinearity of the wave evolution equations, there is no uniform approach to obtain all solutions of the nonlinear wave evolution system. Hence, to find more precise and more effective methods for acquiring the nonlinear wave evolution equations has been an attractive research business. In the last few decades, quite a number of research work has been designed to research various types of nonlinear wave evolution equations. They include effective and broadly applicable techniques such as the finite difference method, variational iteration method, finite element method, finite volume method, boundary elements method, etc.

In recent years, lattice Boltzmann (LB) method has been developed as an optional numerical method to study nonlinear wave propagate equations and evolution of complexity physical system [3,4], especially in liquid mechanics [5,6,7,8]. Unlike more conventional numerical approaches, which are based on the discretization of macroscopic evolution equations, the LB method is based on the mesoscopic kinetic Boltzmann equations for discrete distribution functions. The basic viewpoint is to substitute the macroscopic hydrodynamic equations by a simplified mesoscopic equation modeled on the kinetic theory of gases. To get the hydrodynamic quantities, such as velocity, temperature, pressure, we use the Chapman-Enskog (C-E) multiscale expansion [9] which exploits a small parameter approximation to depict slowly varying solutions of the underlying kinetic evolution equations. This mesoscopic kinetic method has wide prospects in different areas, such as particle suspensions [10], approximate incompressible flows [11,12,13,14], compressible flows [15,16,17,18,19,20,21,22,23,24,25,26,27,28], biofilter media [29], and thermal multiphase flows [30]. Recently, the LB method has been successfully extended to some simulations of NPDEs, including the Korteweg-de Vries equation [31], the Gross-Pitaevskii equation [32], the convection-diffusion equation [33,34,35,36,37,38,39], the Poisson equation [40,41], the Kuramoto-Sivashinsky equation [42], the wave equation [43,44,45,46], the Dirac equation [47], etc. From the point view of calculation, its remarkable advantages include inherent parallelism, geometrical flexibility, numerical efficiency, simplicity of programming and simplicity in dealing with complex boundary conditions.

In this work, we consider the two-component system of coupled sine-Gordon equations, which was introduced recently by Khusnutdinova and Pelinovsky [48]. The basic one-dimensional form is shown as follows:

(1) $$\left\{\begin{array}{c} \frac{\partial^{2}u}{\partial t^{2}}-\frac{\partial^{2}u}{\partial x^{2}}=-\delta^{2}\sin\left(u-w\right),\\ \frac{\partial^{2}w}{\partial t^{2}}-\alpha^{2}\frac{\partial^{2}w}{\partial x^{2}}=\sin\left(u-w\right), \end{array}\left(\alpha >0\;\mathrm{and}\;\delta >0\right)\right.$$

where α remarks the ratio of the acoustic velocities between the components *u* and *w*, the dimensionless parameter $\delta^{2}$ is the same with the ratio of masses of particles in the “lower” and the “upper” parts of the crystal. This system produces the Frenkel-Kontorova dislocation model [49], and this system has also turned out to be highly suitable to describe fluxon phenomena of stacked intrinsic Josephson junctions in high temperature superconductors. Moreover, this system has been studied extensively for two-junction stacks, for stacks consisting of more junctions only some special cases have been analyzed [50]. In addition, this system (1) with α=1 was proposed to describe the open states in DNA [51].

We consider the above system (1) with the initial conditions as follows:

(2) $$\begin{array}{c} \left\{\begin{array}{c} u\left(x,t_{0}\right)=\varphi_{1}\left(x\right),\;x\in \Omega ,\\ \frac{\partial u(x,t_{0})}{\partial t}=\psi_{1}\left(x\right),\;x\in \Omega ,\\ w\left(x,t_{0}\right)=\varphi_{2}\left(x\right),\;x\in \Omega ,\\ \frac{\partial w(x,t_{0})}{\partial t}=\psi_{2}\left(x\right),\;x\in \Omega , \end{array}\right. \end{array}$$

and the boundary conditions:

(3) $$\begin{array}{c} \left\{\begin{array}{c} u\left(a,t\right)=\phi_{1}\left(t\right),\;t\geq t_{0},\\ u\left(b,t\right)=\phi_{2}\left(t\right),\;t\geq t_{0},\\ w\left(a,t\right)=\phi_{3}\left(t\right),\;t\geq t_{0},\\ w\left(b,t\right)=\phi_{4}\left(t\right),\;t\geq t_{0}, \end{array}\right. \end{array}$$

where $\varphi_{1}\left(x\right)$, $\psi_{1}\left(x\right)$, $\varphi_{2}\left(x\right)$, $\psi_{2}\left(x\right)$ and $\phi_{i}\left(t\right)\left(i=1,2,3,4\right)$ are known functions.

There are many analytical methods solving the two-component system of coupled sine-Gordon equations, such as the modified decomposition method [52], the homotopy analysis method [53], the hyperbolic auxiliary function method [54], the homotopy perturbation method [55], the rational exponential ansatz method [56], the variational iteration method [57] and the modified Kudryashov method [58]. However, to our best knowledge, there are few numerical method to solve this coupled system. In recent years, the studies in Refs. [33,59,60] show that the LB method may be an valid numerical solver for real and complex nonlinear coupled systems. Therefore, it is worthy to more study LB method and enlarge its applications. As far as we know, there is no LB model for the two-component system of coupled sine-Gordon equations. The system has similar structure of the convection-diffusion system except for the second time derivative. We can define the first derivatives of macroscopic variable as the sum of distribution functions by the thought of the reference [44]. The main goal of this work is to extend the LB model to solve this two-component system of coupled sine-Gordon numerically by using the double mesoscopic Boltzmann equations. Through the C-E multiscale expansion, the governing nonlinear coupled evolution equations are recovered accurately from the double continuous Boltzmann equations. In order to compare the numerical solutions with the analytic ones, three test problems are taken into account. It is found that the numerical solutions are in accordance with the analytical ones. This demonstrates that the present model is an valid and flexible way for actual application.

The content of this paper is arranged as follows. Next section shows our LB model for the coupled sine-Gordon equations for the two-component system through the present model. Numerical validation is presented in Section 3. Finally, a brief summary is made.

## 2. Lattice Boltzmann Model

In the present model, the three-velocity lattice Bhatnagar-Gross-Krook (BGK) model is used. The directions of the particle discrete velocity are defined as $e_{i},\left(i=0,1,2\right)$:

(4) $$\begin{array}{c} \left[e_{0},\;e_{1},\;e_{2}\right]=\left[0,\;1,\;-1\right]. \end{array}$$

The LB equation with double distribution functions for u(x,t) and w(x,t) are given as follows (s=1,2):

(5) $$\begin{array}{c} f_{si}\left(x+ce_{i}\Delta t,t+\Delta t\right)-f_{si}\left(x,t\right)=-\frac{\Delta t}{\tau_{s}}\left(f_{si}\left(x,t\right)-f_{si}^{\left(0\right)}\left(x,t\right)\right)+\Delta th_{si}\left(x,t\right), \end{array}$$

where $f_{si}\left(x,t\right)$ and $f_{si}^{\left(0\right)}\left(x,t\right)$ refer to the distribution function and equilibrium distribution function, respectively. $h_{si}\left(x,t\right)$ is defined as an amending function, *c* is a constant to determine the viscous coefficient, Δt is the time step, $\tau_{s}$ the dimensionless single-relaxation-time which regulates the rate of approach to the equilibrium. The stability of the equation needs $\tau_{s}>\Delta t/2$ [61].

Unlike the normal LB method, the first derivatives of macroscopic variables u(x,t) and w(x,t) are defined [44] as follows:

(6) $$\left\{\begin{array}{c} \frac{\partial u(x,t)}{\partial t}=\sum_{i}f_{1i}\left(x,t\right),\\ \frac{\partial w(x,t)}{\partial t}=\sum_{i}f_{2i}\left(x,t\right). \end{array}\right.$$

Thus, the steady macroscopical quantities meet the following conservative conditions:

(7) $$\begin{array}{c} \left\{\begin{array}{c} \sum_{i}f_{1i}^{\left(0\right)}\left(x,t\right)=\frac{\partial u(x,t)}{\partial t},\\ \sum_{i}f_{2i}^{\left(0\right)}\left(x,t\right)=\frac{\partial w(x,t)}{\partial t}. \end{array}\right. \end{array}$$

Afterwards, through choosing appropriate local equilibrium distributions and amending functions, the corresponding macroscopic coupled system can be retrieved correctly.

Next, we will give the detailed derivation. Applying the Taylor expansion to the left-hand side of Equation (5) about the point *x* and *t*, we can obtain (s=1,2):

(8) $$\begin{array}{c} \Delta t\left(\frac{\partial }{\partial t}+ce_{i}\frac{\partial }{\partial x}\right)f_{si}+\frac{\Delta t^{2}}{2}{\left(\frac{\partial }{\partial t}+ce_{i}\frac{\partial }{\partial x}\right)}^{2}f_{si}+O\left(\Delta t^{3}\right)=-\frac{\Delta t}{\tau_{s}}\left(f_{si}-f_{si}^{\left(0\right)}\right)+\Delta th_{si}. \end{array}$$

By introducing the C-E multiscale expansions, we can expand the distribution function $f_{si}$ around $f_{si}^{\left(0\right)}$ as follows:

(9) $$\begin{array}{c} \left\{\begin{array}{c} f_{si}=f_{si}^{\left(0\right)}+\varepsilon f_{si}^{\left(1\right)}+\varepsilon^{2}f_{si}^{\left(2\right)}+O\left(\varepsilon^{3}\right),\\ h_{si}=\varepsilon^{2}h_{si}^{\left(1\right)},\\ \frac{\partial }{\partial t}=\varepsilon \frac{\partial }{\partial t_{1}}+\varepsilon^{2}\frac{\partial }{\partial t_{2}},\\ \frac{\partial }{\partial x}=\varepsilon \frac{\partial }{\partial x_{1}}. \end{array}\right. \end{array}$$

And $f_{si}^{\left(k\right)}\left(x,t\right)$ (k=1,2,⋯) are the non-equilibrium distribution functions, which satisfy the solvability conditions (s=1,2):

(10) $$\begin{array}{c} \sum_{i}f_{si}^{\left(k\right)}\left(x,t\right)=0\;\;\left(k=1,2,\cdots \right). \end{array}$$

Dividing both sides of Equation (8) by Δt and substituting Equation (9) into Equation (8), we have:

(11) $$\begin{array}{c} \begin{array}{c} \left(\varepsilon \left(\frac{\partial }{\partial t_{1}}+ce_{i}\frac{\partial }{\partial x_{1}}\right)+\varepsilon^{2}\frac{\partial }{\partial t_{2}}\right)\left(f_{si}^{\left(0\right)}+\varepsilon f_{si}^{\left(1\right)}\right)+\frac{\Delta t}{2}{\left(\varepsilon \left(\frac{\partial }{\partial t_{1}}+ce_{i}\frac{\partial }{\partial x_{1}}\right)+\varepsilon^{2}\frac{\partial }{\partial t_{2}}\right)}^{2}f_{si}^{\left(0\right)}\\ =-\frac{1}{\tau_{s}}\left(\varepsilon f_{si}^{\left(1\right)}+\varepsilon^{2}f_{si}^{\left(2\right)}\right)+\varepsilon^{2}h_{si}^{\left(1\right)}. \end{array} \end{array}$$

Comparing the two sides of Equation (11) and setting terms of order ε to each other, we have O(ε):

(12) $$\left(\frac{\partial }{\partial t_{1}}+ce_{i}\frac{\partial }{\partial x_{1}}\right)f_{si}^{\left(0\right)}=-\frac{1}{\tau_{s}}f_{si}^{\left(1\right)}.$$

Therefore:

(13) $$\begin{array}{c} f_{si}^{\left(1\right)}=-\tau_{s}\left(\frac{\partial }{\partial t_{1}}+ce_{i}\frac{\partial }{\partial x_{1}}\right)f_{si}^{\left(0\right)}. \end{array}$$

Comparing the two sides of Equation (11) and setting terms of order $\varepsilon^{2}$ to each other, we get $O(\varepsilon^{2})$:

(14) $$\begin{array}{c} \frac{\partial }{\partial t_{2}}f_{si}^{\left(0\right)}+\left(\frac{\partial }{\partial t_{1}}+ce_{i}\frac{\partial }{\partial x_{1}}\right)f_{si}^{\left(1\right)}+\frac{\Delta t}{2}{\left(\frac{\partial }{\partial t_{1}}+ce_{i}\frac{\partial }{\partial x_{1}}\right)}^{2}f_{si}^{\left(0\right)}=-\frac{1}{\tau_{s}}f_{si}^{\left(2\right)}+h_{si}^{\left(1\right)}. \end{array}$$

Substituting Equation (13) into Equation (14), we get:

(15) $$\begin{array}{c} \frac{\partial }{\partial t_{2}}f_{si}^{\left(0\right)}+\left(\frac{\Delta t}{2}-\tau_{s}\right){\left(\frac{\partial }{\partial t_{1}}+ce_{i}\frac{\partial }{\partial x_{1}}\right)}^{2}f_{si}^{\left(0\right)}=-\frac{1}{\tau_{s}}f_{si}^{\left(2\right)}+h_{si}^{\left(1\right)}, \end{array}$$

that is:

(16) $$\begin{array}{c} \frac{\partial }{\partial t_{2}}f_{si}^{\left(0\right)}+\left(\frac{\Delta t}{2}-\tau_{s}\right)\left(\frac{\partial^{2}}{\partial t_{1}^{2}}+2ce_{i}\frac{\partial }{\partial t_{1}}\frac{\partial }{\partial x_{1}}+c^{2}e_{i}^{2}\frac{\partial^{2}}{\partial x_{1}^{2}}\right)f_{si}^{\left(0\right)}=-\frac{1}{\tau_{s}}f_{si}^{\left(2\right)}+h_{si}^{\left(1\right)}. \end{array}$$

Summing Equation (12) over *i*, we obtain:

(17) $$\frac{\partial }{\partial t_{1}}\sum_{i}f_{si}^{\left(0\right)}+\frac{\partial }{\partial x_{1}}\sum_{i}ce_{i}f_{si}^{\left(0\right)}=0.$$

Summing Equation (16) over *i*, and using Equation (17), we obtain:

(18) $$\begin{array}{c} \frac{\partial }{\partial t_{2}}\sum_{i}f_{si}^{\left(0\right)}+\left(\frac{\Delta t}{2}-\tau_{s}\right)\left(\frac{\partial }{\partial t_{1}}\frac{\partial }{\partial x_{1}}\sum_{i}ce_{i}f_{si}^{\left(0\right)}+\frac{\partial^{2}}{\partial x_{1}^{2}}\sum_{i}c^{2}e_{i}^{2}f_{si}^{\left(0\right)}\right)=\sum_{i}h_{si}^{\left(1\right)}. \end{array}$$

According to the macroscopic equations, the local equilibrium distribution function $f_{si}^{\left(0\right)}\left(x,t\right)$ is required to satisfy the following relations:

(19) $$\begin{array}{c} \left\{\begin{array}{c} \sum_{i}f_{1i}^{\left(0\right)}\left(x,t\right)=\frac{\partial u}{\partial t},\\ \sum_{i}f_{2i}^{\left(0\right)}\left(x,t\right)=\frac{\partial w}{\partial t},\\ \sum_{i}ce_{i}f_{1i}^{\left(0\right)}\left(x,t\right)=\sum_{i}ce_{i}f_{2i}^{\left(0\right)}\left(x,t\right)=0,\\ \sum_{i}c^{2}e_{i}^{2}f_{1i}^{\left(0\right)}\left(x,t\right)=\mu_{1}u\left(x,t\right),\\ \sum_{i}c^{2}e_{i}^{2}f_{2i}^{\left(0\right)}\left(x,t\right)=\mu_{2}w\left(x,t\right), \end{array}\right. \end{array}$$

in terms of:

(20) $$\begin{array}{c} \mu_{1}=\frac{2}{2\tau_{1}-\Delta t},\;\mu_{2}=\frac{2\alpha^{2}}{2\tau_{2}-\Delta t}. \end{array}$$

Meanwhile, the source term $h_{si}$ satisfies:

(21) $$\begin{array}{c} \left\{\begin{array}{c} \sum_{i}h_{1i}\left(x,t\right)=\sum_{i}\varepsilon^{2}h_{1i}^{\left(1\right)}\left(x,t\right)=-\delta^{2}\sin\left(u-w\right),\\ \sum_{i}h_{2i}\left(x,t\right)=\sum_{i}\varepsilon^{2}h_{2i}^{\left(1\right)}\left(x,t\right)=\sin\left(u-w\right). \end{array}\right. \end{array}$$

With Equation (19), Equation (17) becomes:

(22) $$\frac{\partial^{2}u}{\partial t_{1}\partial t}=0,$$

and:

(23) $$\frac{\partial^{2}w}{\partial t_{1}\partial t}=0.$$

With Equations (19), Equation (18) becomes:

(24) $$\frac{\partial^{2}u}{\partial t_{2}\partial t}+\mu_{1}\left(\frac{\Delta t}{2}-\tau_{1}\right)\frac{\partial^{2}u}{\partial x_{1}^{2}}=\sum_{i}h_{1i}^{\left(1\right)},$$

and:

(25) $$\frac{\partial^{2}w}{\partial t_{2}\partial t}+\mu_{2}\left(\frac{\Delta t}{2}-\tau_{2}\right)\frac{\partial^{2}w}{\partial x_{1}^{2}}=\sum_{i}h_{2i}^{\left(1\right)}.$$

When (22) ×ε + (24) $\times \varepsilon^{2}$ is applied, the final equation is:

(26) $$\begin{array}{c} \frac{\partial^{2}u}{\partial t^{2}}-\frac{\partial^{2}u}{\partial x^{2}}=-\delta^{2}\sin\left(u-w\right). \end{array}$$

When (23) ×ε +(25) $\times \varepsilon^{2}$ is applied, the final equation is:

(27) $$\begin{array}{c} \frac{\partial^{2}w}{\partial t^{2}}-\alpha^{2}\frac{\partial^{2}w}{\partial x^{2}}=\sin\left(u-w\right). \end{array}$$

Meanwhile, from Equation (19), we can get the local equilibrium distribution functions $f_{si}^{\left(0\right)}\left(x,t\right),\left(s=1,2,\;i=0,1,2\right)$ as:

(28) $$\begin{array}{c} \left\{\begin{array}{c} f_{10}^{\left(0\right)}=\frac{\partial u}{\partial t}-\frac{\mu_{1}u}{c^{2}},\\ f_{11}^{\left(0\right)}=f_{12}^{\left(0\right)}=\frac{\mu_{1}u}{2c^{2}},\\ f_{20}^{\left(0\right)}=\frac{\partial w}{\partial t}-\frac{\mu_{2}w}{c^{2}},\\ f_{21}^{\left(0\right)}=f_{22}^{\left(0\right)}=\frac{\mu_{2}w}{2c^{2}}. \end{array}\right. \end{array}$$

From Equation (21), the amending functions $h_{si}\left(x,t\right),\left(s=1,2,\;i=0,1,2\right)$ can be determined. For the sake of simplicity, only one case is presented here:

(29) $$h_{10}\left(x,t\right)=h_{11}\left(x,t\right)=h_{12}\left(x,t\right)=-\frac{\delta^{2}\sin\left(u-w\right)}{3},$$

and:

(30) $$h_{20}\left(x,t\right)=h_{21}\left(x,t\right)=h_{22}\left(x,t\right)=\frac{\sin(u-w)}{3}.$$

In the simulation process, in order to get u(x,t) and w(x,t), we can apply the backward difference to the items $\frac{\partial u(x,t)}{\partial t}$ and $\frac{\partial w(x,t)}{\partial t}$ as:

(31) $$\frac{\partial u(x,t)}{\partial t}=\frac{u(x,t)-u(x,t-\Delta t)}{\Delta t},$$

and:

(32) $$\frac{\partial w(x,t)}{\partial t}=\frac{w(x,t)-w(x,t-\Delta t)}{\Delta t},$$

then using Equation (6), we get:

(33) $$u\left(x,t\right)=\Delta t\sum_{i}f_{1i}\left(x,t\right)+u\left(x,t-\Delta t\right),$$

and:

(34) $$w\left(x,t\right)=\Delta t\sum_{i}f_{2i}\left(x,t\right)+w\left(x,t-\Delta t\right).$$

## 3. Numerical Simulation

In order to test the accuracy and efficiency of the present LB model, three initial and boundary value problems which have analytical solutions are simulated.

For the sake of numerical stability of the finite LB scheme, Δx/Δt≥1 is adopt in all simulations. Initially, the distribution functions $f_{si}\left(x,t\right)$ are set to equal $f_{si}^{\left(0\right)}\left(x,t\right)$. And the macroscopic variables u(x,t) and w(x,t) in Equation (1) are set to equal the initial conditions. The initial and boundary conditions of the test problems with analytical solutions are content with their analytical solutions. The non-equilibrium extrapolation scheme [62] is adopted to deal with the boundary condition.

Firstly, let us introduce symbols $f_{s,i,j}^{n}=f_{si}\left(x_{j},t_{n}\right),\left(s=1,2,\;i=0,1,2\right)$, $u_{j}^{n}=u\left(x_{j},t_{n}\right)$, $w_{j}^{n}=w\left(x_{j},t_{n}\right)$, $x_{j}=j\Delta x$, $t_{n}=n\Delta t$, *n* is the *n*th layer time, *j* is the spatial grid. Then we can reformulate the LB Equation (5) by the classical finite difference notation:

(35) $$\begin{array}{c} \left\{\begin{array}{c} f_{1,0,j}^{n+1}=\left(1-\frac{\Delta t}{\tau_{1}}\right)f_{1,0,j}^{n}+\frac{\Delta t}{\tau_{1}}\left(f_{1,0,j}^{n}+f_{1,1,j}^{n}+f_{1,2,j}^{n}-\frac{\mu_{1}}{c^{2}}u_{j}^{n}\right)-\frac{\delta^{2}\Delta t}{3}\sin\left(u_{j}^{n}-w_{j}^{n}\right),\\ f_{1,1,j+1}^{n+1}=\left(1-\frac{\Delta t}{\tau_{1}}\right)f_{1,1,j}^{n}+\frac{\Delta t}{\tau_{1}}\frac{\mu_{1}}{2c^{2}}u_{j}^{n}-\frac{\delta^{2}\Delta t}{3}\sin\left(u_{j}^{n}-w_{j}^{n}\right),\\ f_{1,2,j-1}^{n+1}=\left(1-\frac{\Delta t}{\tau_{1}}\right)f_{1,2,j}^{n}+\frac{\Delta t}{\tau_{1}}\frac{\mu_{1}}{2c^{2}}u_{j}^{n}-\frac{\delta^{2}\Delta t}{3}\sin\left(u_{j}^{n}-w_{j}^{n}\right),\\ f_{2,0,j}^{n+1}=\left(1-\frac{\Delta t}{\tau_{2}}\right)f_{2,0,j}^{n}+\frac{\Delta t}{\tau_{2}}\left(f_{2,0,j}^{n}+f_{2,1,j}^{n}+f_{2,2,j}^{n}-\frac{\mu_{2}}{c^{2}}w_{j}^{n}\right)+\frac{\Delta t}{3}\sin\left(u_{j}^{n}-w_{j}^{n}\right),\\ f_{2,1,j+1}^{n+1}=\left(1-\frac{\Delta t}{\tau_{2}}\right)f_{2,1,j}^{n}+\frac{\Delta t}{\tau_{2}}\frac{\mu_{2}}{2c^{2}}w_{j}^{n}+\frac{\Delta t}{3}\sin\left(u_{j}^{n}-w_{j}^{n}\right),\\ f_{2,2,j-1}^{n+1}=\left(1-\frac{\Delta t}{\tau_{2}}\right)f_{2,2,j}^{n}+\frac{\Delta t}{\tau_{2}}\frac{\mu_{2}}{2c^{2}}w_{j}^{n}+\frac{\Delta t}{3}\sin\left(u_{j}^{n}-w_{j}^{n}\right). \end{array}\right. \end{array}$$

At time (n+1)Δt, *u* and *w* are updated as follows:

(36) $$u_{j}^{n+1}=\Delta t\left(f_{1,0,j}^{n+1}+f_{1,1,j}^{n+1}+f_{1,2,j}^{n+1}\right)+u_{j}^{n},$$

and:

(37) $$w_{j}^{n+1}=\Delta t\left(f_{2,0,j}^{n+1}+f_{2,1,j}^{n+1}+f_{2,2,j}^{n+1}\right)+w_{j}^{n}.$$

The initial local equilibrium distribution functions $f_{s,i,j}^{0},\left(s=1,2,\;i=0,1,2\right)$ are:

(38) $$\begin{array}{c} \left\{\begin{array}{c} f_{1,0,j}^{0}={\left(\frac{\partial u}{\partial t}\right)}_{j}^{0}-\frac{\mu_{1}}{c^{2}}u_{j}^{0},\\ f_{1,1,j}^{0}=f_{1,2,j}^{0}=\frac{\mu_{1}}{2c^{2}}u_{j}^{0},\\ f_{2,0,j}^{0}={\left(\frac{\partial w}{\partial t}\right)}_{j}^{0}-\frac{\mu_{2}}{c^{2}}w_{j}^{0},\\ f_{2,1,j}^{0}=f_{2,2,j}^{0}=\frac{\mu_{2}}{2c^{2}}w_{j}^{0}, \end{array}\right. \end{array}$$

where $u_{j}^{0}=u\left(x_{j},t_{0}\right)$, $w_{j}^{0}=w\left(x_{j},t_{0}\right)$, ${\left(\frac{\partial u}{\partial t}\right)}_{j}^{0}=\frac{\partial u(x_{j},t_{0})}{\partial t}$ and ${\left(\frac{\partial w}{\partial t}\right)}_{j}^{0}=\frac{\partial w(x_{j},t_{0})}{\partial t}$.

The global relative error (GRE) is introduced to measure the present model’s precision, and defined as follows:

(39) $$\begin{array}{c} \mathrm{GRE}=\frac{\sum_{j=0}^{N}\left|u\left(x_{j},t\right)-u^{*}\left(x_{j},t\right)\right|}{\sum_{j=0}^{N}\left|u^{*}\left(x_{j},t\right)\right|}, \end{array}$$

where $u(x_{j},t)$, $u^{*}\left(x_{j},t\right)$ represent the numerical solution and analytical one, respectively. The summation is taken all grid points together. Next, numerical tests are performed for different initial conditions of the coupled sine-Gordon equations. It is found that the numerical solutions are in accordance with the analytical solutions over a relatively long period of time.

**Example** **1.** *Consider the two-component system of coupled sine-Gordon equations in the region −5≤x≤5 given as:*

(40) $$\left\{\begin{array}{c} \frac{\partial^{2}u}{\partial t^{2}}-\frac{\partial^{2}u}{\partial x^{2}}=-\delta^{2}\sin\left(u-w\right),\\ \frac{\partial^{2}w}{\partial t^{2}}-\alpha^{2}\frac{\partial^{2}w}{\partial x^{2}}=\sin\left(u-w\right), \end{array}\right.$$

*with the initial conditions:*

(41) $$\begin{array}{c} \left\{\begin{array}{c} u\left(x,0\right)=\frac{\delta^{2}}{4\left(b^{2}-1\right)\mu^{2}}\sin\left(2\mu x\right)+c_{1}\mu x+c_{2},\\ \frac{\partial u}{\partial t}\left(x,0\right)=-bc_{1}\mu -\frac{2b\delta^{2}\cos\left(2\mu x\right)}{4\mu (b^{2}-1)},\\ w\left(x,0\right)=\frac{\delta^{2}}{4\left(b^{2}-1\right)\mu^{2}}\sin\left(2\mu x\right)+c_{1}\mu x+c_{2}-2\arctan\left(\tan\left(\mu x\right)\right),\\ \frac{\partial w}{\partial t}\left(x,0\right)=2b\mu -bc_{1}\mu -\frac{2b\delta^{2}\cos\left(2\mu x\right)}{4\mu (b^{2}-1)}, \end{array}\right. \end{array}$$

*and the analytical solution for this problem is extracted from Ref. [56] by:*

(42) $$\begin{array}{c} \left\{\begin{array}{c} u\left(x,t\right)=\frac{\delta^{2}}{4\left(b^{2}-1\right)\mu^{2}}\sin\left(2\xi \right)+c_{1}\xi +c_{2},\\ w\left(x,t\right)=\frac{\delta^{2}}{4\left(b^{2}-1\right)\mu^{2}}\sin\left(2\xi \right)+c_{1}\xi +c_{2}-2\arctan\left(\tan\left(\xi \right)\right),\\ \xi =\mu (x-bt), \end{array}\right. \end{array}$$

*where $b=\sqrt{\left(1-\alpha^{2}\delta^{2}\right)/\left(1-\delta^{2}\right)}$. The boundary conditions conform to the analytical solution.*

**Figure 1:**
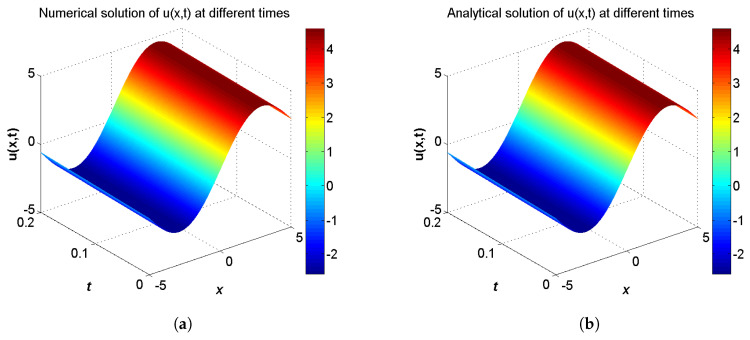
Spatiotemporal evolution graph of the numerical (**a**) and analytical (**b**) solutions up to t=0.2 s, with $N_{x}\times N_{t}=800\times 320$ for Example 1.

**Figure 2:**
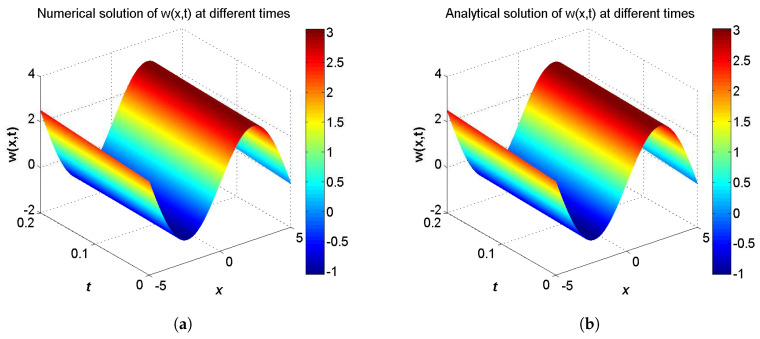
Spatiotemporal evolution graph of the numerical (**a**) and analytical (**b**) solutions up to t=0.2 s, with $N_{x}\times N_{t}=800\times 320$ for Example 1.

**Figure 3:**
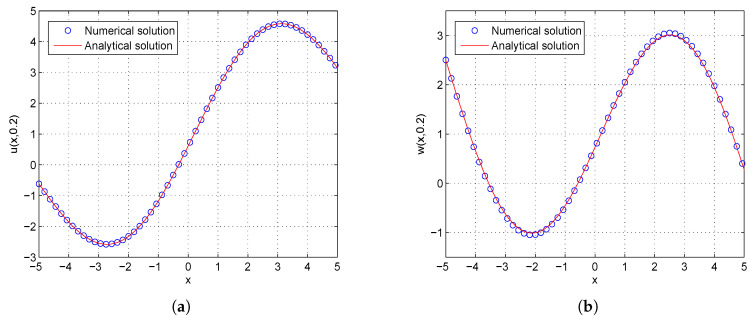
Comparison between numerical and analytical solutions of u(x,t) (**a**) and w(x,t) (**b**) at t=0.2 with $N_{x}\times N_{t}=800\times 320$ for Example 1. The blue circle symbol represents the numerical solution, and the solid red line represents the analytical solution given by Equation (42).

In the proceeding, we adopt α=0.01, δ=0.1, μ=0.3, $c_{1}=c_{2}=1.0$, $\tau_{1}=\tau_{2}=\Delta t$. The computational region is fixed on I=[−5,5]. The global relative errors (GRE) for the solutions u(x,t) and w(x,t) at t=0.2 with different resolutions, from Δx/Δt=10 to 80, and the space grid *N* from 400 to 3200, are listed in Table 1 and Table 2. From these two tables, we can see that GREs for u(x,t) are found to range from $9.9057\times 10^{-7}$ to $6.3625\times 10^{-5}$, and GREs for w(x,t) are found to range from $1.8355\times 10^{-2}$ to $1.8455\times 10^{-2}$. We can also see that when Δx/Δt is larger, namely Δt is relatively smaller, the global relative error of u(x,t) reduces with the first-order accuracy, while the global relative error of w(x,t) changes little. The accuracy of the macroscopic variable w(x,t) is not affected by the resolution in space and time. That is due to the treatment of the items ∂u(x,t)/∂t and ∂w(x,t)/∂t in Equations (31) and (32), has the first-order accuracy. According to Table 1 and Table 2, we adopt $N_{x}\times N_{t}=800\times 320$ in consideration of both the computational accuracy and efficiency. It can be found that the numerical results in according with the analytical solutions, which are presented as the spatiotemporal evolution graph of the numerical (left) and analytical (right) solutions for comparison, see Figure 1 and Figure 2. For clarity of contrast, we also present the two-dimensional visual comparisons of u(x,t) (left) and w(x,t) (right) at t=0.2, see Figure 3. It is evident that the numerical results coincide with the analytical solutions.

**Example** **2.** *Consider the two-component system of coupled sine-Gordon equations in the region −10≤x≤10 given by:*

(43) $$\left\{\begin{array}{c} \frac{\partial^{2}u}{\partial t^{2}}-\frac{\partial^{2}u}{\partial x^{2}}=-\delta^{2}\sin\left(u-w\right),\\ \frac{\partial^{2}w}{\partial t^{2}}-\alpha^{2}\frac{\partial^{2}w}{\partial x^{2}}=\sin\left(u-w\right), \end{array}\right.$$

*with the discontinuous initial conditions:*

(44) $$\begin{array}{c} \left\{\begin{array}{c} u\left(x,0\right)=\frac{\delta^{2}}{4\left(b^{2}-1\right)\mu^{2}}\sin\left(2\mu x\right)+c_{1}\mu x+c_{2},\\ \frac{\partial u}{\partial t}\left(x,0\right)=-bc_{1}\mu -\frac{2b\delta^{2}\cos\left(2\mu x\right)}{4\mu (b^{2}-1)},\\ w\left(x,0\right)=\frac{\delta^{2}}{4\left(b^{2}-1\right)\mu^{2}}\sin\left(2\mu x\right)+c_{1}\mu x+c_{2}-2\arctan\left(\cot\left(\mu x\right)\right),\\ \frac{\partial w}{\partial t}\left(x,0\right)=-2b\mu -bc_{1}\mu -\frac{2b\delta^{2}\cos\left(2\mu x\right)}{4\mu (b^{2}-1)}, \end{array}\right. \end{array}$$

*and the analytical solution for this problem is extracted from Reference [56] by:*

(45) $$\begin{array}{c} \left\{\begin{array}{c} u\left(x,t\right)=\frac{\delta^{2}}{4\left(b^{2}-1\right)\mu^{2}}\sin\left(2\xi \right)+c_{1}\xi +c_{2},\\ w\left(x,t\right)=\frac{\delta^{2}}{4\left(b^{2}-1\right)\mu^{2}}\sin\left(2\xi \right)+c_{1}\xi +c_{2}-2\arctan\left(\cot\left(\xi \right)\right),\\ \xi =\mu (x-bt), \end{array}\right. \end{array}$$

*where $b=\sqrt{\left(1-\alpha^{2}\delta^{2}\right)/\left(1-\delta^{2}\right)}$. The boundary conditions conform to the analytical solution.*

**Figure 4:**
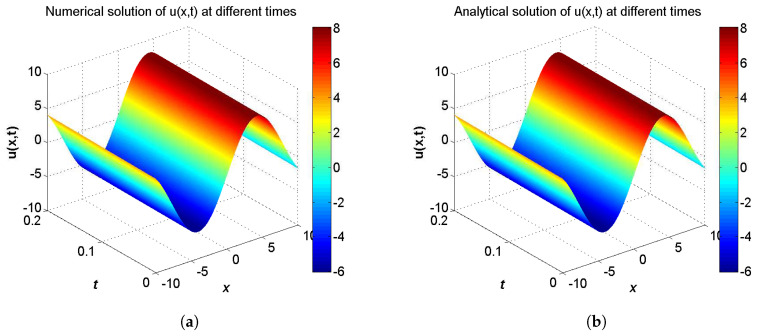
Spatiotemporal evolution graph of the numerical (**a**) and analytical (**b**) solutions up to t = 0.2 s, with $N_{x}\times N_{t}=800\times 320$ for Example 2.

**Figure 5:**
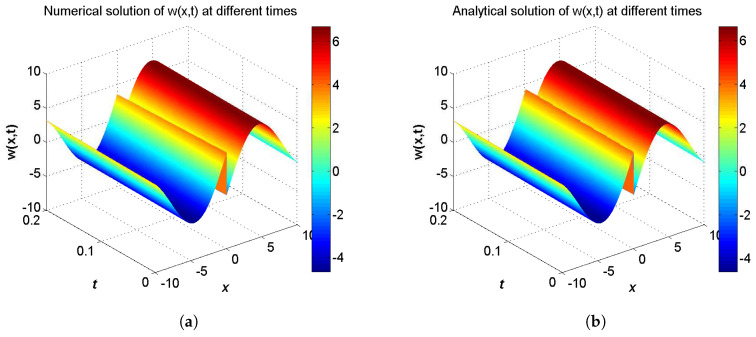
Spatiotemporal evolution graph of the numerical (**a**) and analytical (**b**) solutions up to t = 0.2 s, with $N_{x}\times N_{t}=800\times 320$ for Example 2.

**Figure 6:**
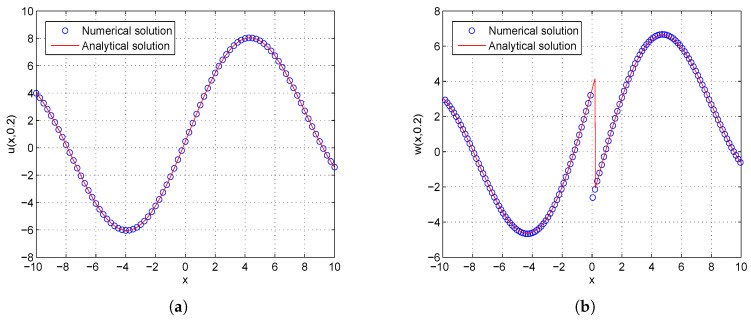
Comparison between numerical and analytical solutions of u(x,t) (**a**) and w(x,t) (**b**) at t=0.2 with $N_{x}\times N_{t}=800\times 320$ for Example 2. The blue circle symbol represents the numerical solution, and the solid red line represents the analytical solution given by Equation (45).

In the proceeding, we adopt α=0.01, δ=0.05, μ=0.2, $c_{1}=c_{2}=1.0$, $\tau_{1}=\tau_{2}=\Delta t$. The computational region is fixed within I=[−10,10]. The GREs for the solutions u(x,t) and w(x,t) at t=0.2 in different resolutions, from Δx/Δt=10 to 80 and the space grid *N* from 400 to 3200, are listed in Table 3 and Table 4. From these two tables, we can see that the GREs for u(x,t) range from $1.0980\times 10^{-6}$ to $7.0536\times 10^{-5}$, and the GREs for w(x,t) range from $2.8141\times 10^{-2}$ to $3.2528\times 10^{-2}$. When Δx/Δt is larger, namely Δt is relatively small, the GREs of u(x,t) reduces with first-order accuracy, the GREs of u(x,t) change little. At the same time, we present the spatiotemporal evolution graph of the numerical (left) and analytical (right) solutions of u(x,t) and w(x,t) for comparison, see Figure 4 and Figure 5. For clarity of contrast, we also present the two-dimensional visual comparisons of u(x,t) (left) and w(x,t) (right) at t=0.2, see Figure 6. It can be found that the numerical results in according with the analytical solutions.

**Example** **3.** *Consider the two-component system of coupled sine-Gordon equations in the region −10≤x≤10 given by:*

(46) $$\left\{\begin{array}{c} \frac{\partial^{2}u}{\partial t^{2}}-\frac{\partial^{2}u}{\partial x^{2}}=-\delta^{2}\sin\left(u-w\right),\\ \frac{\partial^{2}w}{\partial t^{2}}-\alpha^{2}\frac{\partial^{2}w}{\partial x^{2}}=\sin\left(u-w\right), \end{array}\right.$$

*with the initial conditions:*

(47) $$\begin{array}{c} \left\{\begin{array}{c} u\left(x,0\right)=-\frac{4\delta^{2}}{\left(b^{2}-1\right)\mu^{2}}arccot\left(2k\exp\left(-\mu x\right)\right)+c_{1}\mu x+c_{2},\\ \frac{\partial u}{\partial t}\left(x,0\right)=\frac{8b\delta^{2}k\exp\left(\mu x\right)}{\mu \left(b^{2}-1\right)\left(4k^{2}+\exp\left(2\mu x\right)\right)}-bc_{1}\mu ,\\ w\left(x,0\right)=-\frac{4\delta^{2}}{\left(b^{2}-1\right)\mu^{2}}arccot\left(2k\exp\left(-\mu x\right)\right)+c_{1}\mu x+c_{2}+2\arctan\left(\frac{1}{4k}\left(\exp\left(\mu x\right)-4k^{2}\exp\left(-\mu x\right)\right)\right),\\ \frac{\partial w}{\partial t}\left(x,0\right)=-\frac{8k^{2}\exp\left(\mu x\right)\left(4b\mu k^{2}+b\mu \exp\left(2\mu x\right)\right)}{{\left(\exp\left(2\mu x\right)-4k^{2}\right)}^{2}+16k^{2}\exp\left(2\mu x\right)}+\frac{8b\delta^{2}k\exp\left(\mu x\right)}{\mu \left(b^{2}-1\right)\left(4k^{2}+\exp\left(2\mu x\right)\right)}-bc_{1}\mu , \end{array}\right. \end{array}$$

*and the analytical solution for this problem is extracted from Reference [56] by:*

(48) $$\begin{array}{c} \left\{\begin{array}{c} u\left(x,t\right)=-\frac{4\delta^{2}}{\left(b^{2}-1\right)\mu^{2}}arccot\left(2k\exp\left(-\xi \right)\right)+c_{1}\xi +c_{2},\\ w\left(x,t\right)=-\frac{4\delta^{2}}{\left(b^{2}-1\right)\mu^{2}}arccot\left(2k\exp\left(-\xi \right)\right)+c_{1}\xi +c_{2}+2\arctan\left(\frac{1}{4k}\left(\exp\left(\xi \right)-4k^{2}\exp\left(-\xi \right)\right)\right),\\ \xi =\mu (x-bt), \end{array}\right. \end{array}$$

*where $\mu =\sqrt{\frac{b^{2}\left(\delta^{2}-1\right)+\left(1-\alpha^{2}\delta^{2}\right)}{\left(b^{2}-1\right)\left(b^{2}-\alpha^{2}\right)}}$. The boundary conditions conform to the analytical solution.*

**Figure 7:**
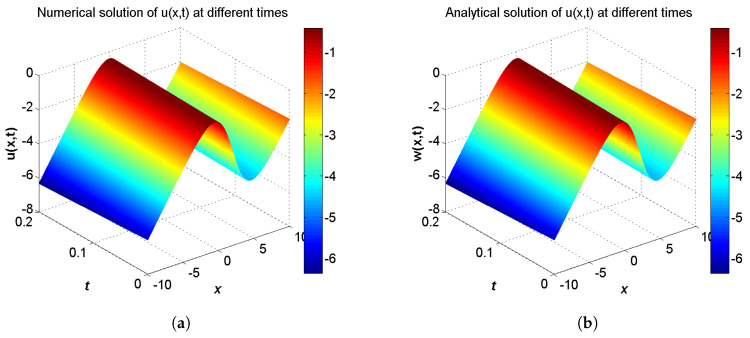
Spatiotemporal evolution graph of the numerical (**a**) and analytical (**b**) solutions up to t=0.2 s, with $N_{x}\times N_{t}=800\times 320$ for Example 3.

**Figure 8:**
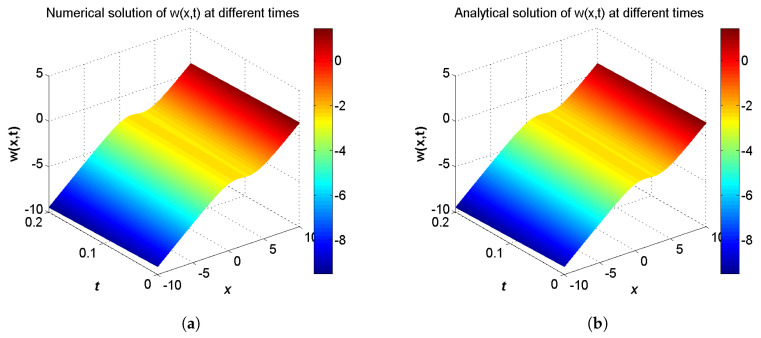
Spatiotemporal evolution graph of the numerical (**a**) and analytical (**b**) solutions up to t=0.2 s, with $N_{x}\times N_{t}=800\times 320$ for Example 3.

**Figure 9:**
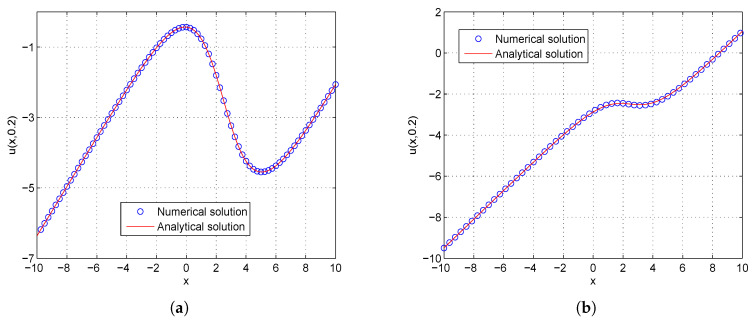
Comparison between numerical and analytical solutions of u(x,t) (**a**) and w(x,t) (**b**) at t=0.2 with $N_{x}\times N_{t}=800\times 320$ for Example 3. The blue circle symbol represents the numerical solution, and the solid red line represents the analytical solution given by Equation (48).

In the proceeding, we adopt α=1.6, δ=2.0, b=2.5, $c_{1}=c_{2}=1.0$, $\tau_{1}=\tau_{2}=\Delta t$. The computational region is fixed within I=[−10,10]. The GREs for the solutions u(x,t) and w(x,t) at t=0.2 in different resolutions, from Δx/Δt=10 to 80 and the space grid *N* from 400 to 3200, which are listed in Table 5 and Table 6. From these two tables, we can see that the GREs for u(x,t) are found to range from $3.2169\times 10^{-5}$ to $2.3832\times 10^{-4}$, and the GREs for w(x,t) are found to range from $2.9026\times 10^{-3}$ to $2.9126\times 10^{-3}$. When Δx/Δt is larger, namely Δt is relatively small, the global relative error of u(x,t) reduces with first-order accuracy, the GREs of u(x,t) changes little. At the same time, we present the spatiotemporal evolution graph of the numerical (left) and analytical (right) solutions for comparison, see Figure 7 and Figure 8. For clarity of contrast, we also present the two-dimensional visual comparisons of u(x,t) (left) and w(x,t) (right) at t=0.2, see Figure 9. It can be found that the numerical results in according with the analytical solutions.

## 4. Conclusions

In conclusion, we have researched the application of the LB method for the solution of the two-component system of coupled sine-Gordon equations. By choosing the equilibrium distribution function and an amending function suitably, according to our proposed model, the governing evolution equations can be recovered accurately, in which the Chapman-Enskog multiscale expansion is employed. Numerical simulation for three test problems has been conducted to validate the LB model. The numerical results are in good agreement with the analytical solutions. While we take different initial conditions, we can get unique numerical solutions. We can also see that when Δx/Δt is larger, Δt is relatively smaller, and the global relative error of u(x,t) reduces with first-order accuracy; nevertheless, the global relative error of w(x,t) changes little. It is found that the accuracy of the macroscopic variable w(x,t) is not affected by the resolution in space and time. That is due to the treatment of the items ∂u(x,t)/∂t and ∂w(x,t)/∂t in Equations (31) and (32), which have first-order accuracy. For the purpose of attaining better computational accuracy and efficiency, the LB method for the test problems needs relatively small time step and space step. The present model can be developed to research more different types of the nonlinear system problems. There are still many problems worth studying to develop the present method, such as how to improve the accuracy and stability; we will continue these study in the near feature.

## Figures and Tables

**Table 1:** The global relative error (GRE) for u(x,0.2) with different Δx/Δt.

Grid *N* (*x*)	Δx/Δt=10	Δx/Δt=20	Δx/Δt=40	Δx/Δt=80
400	$6.3625\times 10^{-5}$	$3.1754\times 10^{-5}$	$1.5831\times 10^{-5}$	$7.8660\times 10^{-6}$
800	$3.1872\times 10^{-5}$	$1.5919\times 10^{-5}$	$7.9393\times 10^{-6}$	$3.9580\times 10^{-6}$
1600	$1.5946\times 10^{-5}$	$7.9671\times 10^{-6}$	$3.9798\times 10^{-6}$	$1.9898\times 10^{-6}$
3200	$7.9738\times 10^{-6}$	$3.9847\times 10^{-6}$	$1.9927\times 10^{-6}$	$9.9057\times 10^{-7}$

**Table 2:** The global relative error (GRE) for w(x,0.2) with different Δx/Δt.

Grid *N* (*x*)	Δx/Δt=10	Δx/Δt=20	Δx/Δt=40	Δx/Δt=80
400	$1.8455\times 10^{-2}$	$1.8398\times 10^{-2}$	$1.8369\times 10^{-2}$	$1.8355\times 10^{-2}$
800	$1.8425\times 10^{-2}$	$1.8397\times 10^{-2}$	$1.8382\times 10^{-2}$	$1.8375\times 10^{-2}$
1600	$1.8410\times 10^{-2}$	$1.8396\times 10^{-2}$	$1.8389\times 10^{-2}$	$1.8385\times 10^{-2}$
3200	$1.8403\times 10^{-2}$	$1.8395\times 10^{-2}$	$1.8392\times 10^{-2}$	$1.8390\times 10^{-2}$

**Table 3:** The global relative error (GRE) for u(x,0.2) with different Δx/Δt.

Grid *N* (*x*)	Δx/Δt=10	Δx/Δt=20	Δx/Δt=40	Δx/Δt=80
400	$7.0536\times 10^{-5}$	$3.5222\times 10^{-5}$	$1.7555\times 10^{-5}$	$8.7270\times 10^{-6}$
800	$3.5327\times 10^{-5}$	$1.7652\times 10^{-5}$	$8.8120\times 10^{-6}$	$4.3904\times 10^{-6}$
1600	$1.7674\times 10^{-5}$	$8.8345\times 10^{-6}$	$4.4097\times 10^{-6}$	$2.1992\times 10^{-6}$
3200	$8.8383\times 10^{-6}$	$4.4151\times 10^{-6}$	$2.2029\times 10^{-6}$	$1.0980\times 10^{-6}$

**Table 4:** The global relative error (GRE) for w(x,0.2) with different Δx/Δt.

Grid *N* (*x*)	Δx/Δt=10	Δx/Δt=20	Δx/Δt=40	Δx/Δt=80
400	$3.2528\times 10^{-2}$	$3.2482\times 10^{-2}$	$3.2459\times 10^{-2}$	$3.2447\times 10^{-2}$
800	$3.0029\times 10^{-2}$	$3.0006\times 10^{-2}$	$2.9994\times 10^{-2}$	$2.9988\times 10^{-2}$
1600	$2.8777\times 10^{-2}$	$2.8766\times 10^{-2}$	$2.8760\times 10^{-2}$	$2.8757\times 10^{-2}$
3200	$2.8151\times 10^{-2}$	$2.8145\times 10^{-2}$	$2.8142\times 10^{-2}$	$2.8141\times 10^{-2}$

**Table 5:** The global relative error (GRE) for u(x,0.2) with different Δx/Δt.

Grid *N* (*x*)	Δx/Δt=10	Δx/Δt=20	Δx/Δt=40	Δx/Δt=80
400	$2.3832\times 10^{-4}$	$1.2610\times 10^{-4}$	$7.0571\times 10^{-5}$	$4.7861\times 10^{-5}$
800	$1.2849\times 10^{-4}$	$7.2477\times 10^{-5}$	$4.7318\times 10^{-5}$	$3.8168\times 10^{-5}$
1600	$7.3027\times 10^{-5}$	$4.7296\times 10^{-5}$	$3.7867\times 10^{-5}$	$3.3993\times 10^{-5}$
3200	$4.7310\times 10^{-5}$	$3.7804\times 10^{-5}$	$3.3898\times 10^{-5}$	$3.2169\times 10^{-5}$

**Table 6:** The global relative error (GRE) for w(x,0.2) with different Δx/Δt.

Grid *N* (*x*)	Δx/Δt=10	Δx/Δt=20	Δx/Δt=40	Δx/Δt=80
400	$2.9126\times 10^{-3}$	$2.9069\times 10^{-3}$	$2.9040\times 10^{-3}$	$2.9026\times 10^{-3}$
800	$2.9124\times 10^{-3}$	$2.9095\times 10^{-3}$	$2.9081\times 10^{-3}$	$2.9073\times 10^{-3}$
1600	$2.9121\times 10^{-3}$	$2.9107\times 10^{-3}$	$2.9099\times 10^{-3}$	$2.9096\times 10^{-3}$
3200	$2.9119\times 10^{-3}$	$2.9112\times 10^{-3}$	$2.9108\times 10^{-3}$	$2.9107\times 10^{-3}$
